# Supplementary material for: PEMT rs7946 Polymorphism and Sex Modify the Effect of Adequate Dietary Choline Intake on the Risk of Hepatic Steatosis in Older Patients with Metabolic Disorders
Source: Nutrients. 2023 Jul 19;15(14):3211. doi: 10.3390/nu15143211 (PMC10383596; doi:10.3390/nu15143211)
Supplement: Supplementary file 1 [file nutrients-15-03211-s001.zip › nutrients-2491279-supplementary.pdf]

Table S1 Demographic data and genetic polymorphism status in hepatic steatois (HS) and control groups.

Variables	HS	Control	P value
No.	152	98	
Male, No. (%)	92(60.5)	45(45.9)	0.023
Age, yr	60(53-66)	63(55-68)	0.083
BMI, kg/m^2	27.2(24.4-30.4)	22.9(20.1-25.0)	<0.001
Smoking, No. (%)	13(11.2))	7(7.1)	0.290
Alcohol, , No. (%)	20(13.2)	12(12.2)	0.833
Biochemical markers			
Total cholesterol (mg/dL)	193(170-217)	197(170-224)	0.437
Triglycerides (md/dL)	130(90-194)	72(53-93.3))	<0.001
LDL (mg/dL)	111(91-137)	110(86-133)	0.432
HDL (mg/dL)	41.0(36.0-48.0)	55.0(46.0-66.0)	<0.001
AST (U/L)	29.0(22.0-41.0)	23.0(19.0-26.3)	<0.001
ALT (U/L)	37.0 (26.0-60.8)	19.0 (10.0-25.0)	<0.001
GGT (U/L)	27.0(19.0-48.0)	15.0(12.0-23.0)	<0.001
CRE (mg/dL)	0.86(0.67-1.02)	0.73(0.63-0.91)	0.003
CPK (U/L)	120(82.3-167)	91.5(73.5-136)	0.004
Plasma glucose (mg/dL)	105(98.0-118)	98.0(94.0-115))	<0.001
HbAlc (%)	6.00 (5.70-6.40)	5.60(5.50-6.00)	<0.001
Insulin (uIU/ml)	13.2 (10.1-20.0)	8.02(6.50-10.2)	<0.001
HOMA-IR	3.65 (2.48-5.74)	1.97 (1.62-2.66)	<0.001
Genetic Polymorphism			
PEMT V175M (G→A),	HS, N (%)	Control, N (%)	<0.001
GG	108(71.1)	47(48.0)	
GA	38(25.0)	31(31.6)	
AA	6(3.9)	20(20.4)	

1.Continuous variables were presented by median with the IQR (interquartile range)

2.Continuous variables were compared using the Mann–Whitney U test. Discrete variables were compared using the Chi-square test

3. Differences were considered to be statistically significant at P < 0.05

Table S2: Clinical data on carbon-metabolite status of female participants with PEMT rs7946 (+5464GG) genotype stratified by energy-adjusted choline intake

Choline intake mg/day	**Tertile 1 213-420**	**Tertile 2 420-533**	**Tertile 3 533-914**	**p-value**	**Ptrend**
Patients, n	**23**	**23**	**22**		
Age	67(61-70)	64(55-67)	61.5(53.5-69.3)	0.200	0.068
BMI, kg/m2,	26.3(23.7-29.0)	23.9(22.6-26.7)	24.3(21.7-29.7)	0.132	0.097
Smoking, N(%)	0	0	1(4.5)	0.346	0.212
Alcohol (%)	2(8.7)	3(13.0)	1(4.5)	0.603	0.634
NAFLD	**19(82.6)**	**10(43.5)**	**13(59.1)**	**0.023**	**0.101**
Obesity*	21(91.3)	16(69.6)	17(77.3)	0.181	0.241
Cholesterol	199(164-227)	193(171-222)	201(178-243)	0.765	0.558
**Triglyceride**	**129(87-169)**	**73(56-115)**	**90.5(61.5-138)**	**0.019**	**0.158**

LDL	116(91-146)	110(86-136)	119(90.5-141)	0.852	0.669
**HDL**	**47(38-55)**	**57(46-65)**	**50(42-62.8)**	**0.058**	**0.305**
AST	26(20-44)	23(20-28)	28(24.8-33.5)	0.062	0.370
ALT	33(19-44)	24(19-27)	36(22.8-52.5)	0.047	0.427
GGT	19.0(14.0-31.0)	18.0(12.5-28.8)	29.0(14.8-61.5)	0.135	0.136
Cre	0.66(0.58-0.76)	0.67(0.59-0.72)	0.61(0.54-0.71)	0.555	0.325
**CPK**	**105(75.0-146)**	**100(71.0-145)**	**81.5(57.8-97.3)**	**0.053**	**0.033**
**HbA1C**	**6.2(5.7-6.4)**	**5.8(5.6-6.1)**	**5.75(5.5-6.13)**	**0.079**	**0.036**
Insulin	**12.3(7.97-15.0)**	**9.24(6.7-14.4)**	11.6(8.42-16.2)	0.276	0.866
HOMA-IR	**3.63(2.03-4.34)**	**2.25(1.69-3.92)**	2.90(2.01-4.30)	0.392	0.978
Plasma folate	11(9.52-16.0)	10.8(7.58-15.6)	11.2(6.98-17.8)	0.553	0.536
Plasma choline	10.9(9.36-13.3)	10.7(9.70-12.6)	10.1(8.21-12.2)	0.353	0.210
Plasma betaine	45.1(34.4-51.7)	45.2(39.3-48.2)	36.9(32.9-51.5)	0.417	0.357
Homocysteine	9.82(8.30-12.3)	8.85(7.5-11.0)	8.73(7.38-10.4)	0.402	0.179
Line-1	78.3(747-83.0)	7.4(76.7-82.9)	79.1(76.4-83.8)	0.702	0.427
Energy	1311(1131-1884)	1329(1004-1906)	1335(1203-1846)	0.934	0.942
**Folate**	**568(297-665)**	**558(461-968)**	**792(721-1249)**	**0.006**	**0.002**
**Betaine**	**183(129-243)**	**196(156-320)**	**264(186-381)**	**0.071**	**0.021**
**Choline**	**337(297-405)**	**470(446-500)**	**594(557-865)**	**<0.001**	**<0.001**

1. Discrete variables were presented by number (%) and compared using the Chi-square test. Differences were considered to be statistically significant at P < 0.05 ;
2. Continuous variables were presented by median with the IQR (interquartile range), compared using the Kruskal–Wallis test; the trend test was performed using Jonckheere–Terpstra test. Differences were considered to be statistically significant at P < 0.05
3. *Obesity was defined as at least one of the following features: BMI ≥ 30kg/m2, Waist–Hip ratio >0.85 in female or >0.9 in male, visceral fat level ≥10 of Inbody test, body fat percent >25% in male or >32 in female. Lean was define as none of above features.

Table S3: Clinical data on carbon-metabolite status of male participants with PEMT rs7946 (+5464GG) genotype stratified by energy-adjusted choline intake

Choline intake mg/day	**Tertile 1 219-418**	**Tertile 2 418-506**	**Tertile 3 506-865**	**p-value**	**Ptrend**
Patients, n	**29**	**29**	**29**		
Age	59(54.5-66.0)	61(50-65)	55(43-64)	0.177	0.075
**BMI, kg/m2,**	**24.2(22.6-28.6)**	**29.4(26.9-30.9)**	**27.1(24.5-31.1)**	**0.011**	**0.030**
Smoking, N(%)	6(20.7)	4(3.8)	5(17.2)	0.785	0.730
Alcohol (%)	3(10.3)	5(17.2)	8(27.6)	0.233	0.092
NAFLD	**18(62.1)**	**24(82.8)**	**24(82.8)**	**0.104**	**0.067**
**Obesity***	**17(59.6)**	**26(89.7)**	**20(69.0)**	**0.027**	**0.381**
Cholesterol	184(167-207)	202(166-216)	197(162-217)	0.669	0.438
Triglyceride	**89(66.5-140)**	**119(72-201)**	116(75.5-194)	0.455	0.265
LDL	109(86.5-131)	118(91-142)	105(89-133)	0.651	0.895

HDL	43(36.5-49.5)	40(34.5-45.5)	41(35-48)	0.457	0.522
AST	24(22-32)	29(20.5-48.0)	26(21.5-34.5)	0.919	0.867
ALT	25(18.5-47.0)	45(20.5-75.0)	31(25.5-56.5)	0.260	0.224
GGT	22.0(15.0-32.0)	28.0(18.0-49.5)	24.0(17.5-42.5)	0.259	0.258
CRE	0.99(0.81-1.07)	0.95(0.87-1.11)	0.92(0.81-1.05)	0.424	0.646
CPK	170(95.0-215)	143(97.0-185)	120(88.5-192)	0.863	0.575
HbA1C	6.0(5.55-6.75)	5.8(5.6-6.5)	5.8(5.5-6.3)	0.503	0.250
**Insulin**	**8.74(6.94-14.5)**	**13.8(113-21.0)**	**11.8(8.6-18.0)**	**0.023**	**0.184**
HOMA-IR	3.41(1.72-4.34)	2.38(2.01-15.3)	2.73(1.90-2.64)	0.107	0.415
Plasma Folate	7.84(6.26-11.1)	7.44(5.18-9.57)	8.45(5.39-11.6)	0.619	0.919
Plasma choline	13.1(12.1-14.7)	14.1(11.1-16.6)	13.1(9.72-18.0)	0.525	0.637
Plasma betaine	49.2(39.0-61.5)	48.5(38.6-54.3)	47.5(36.7-60.4)	0.757	0.588
Homocysteine	12.6(9.08-15.2)	13.3(11.8-17.1)	12.5(10.3-13.9)	0.135	0.649
Line-1	82.1(77.8-86.9)	79.9(73.7-84.5)	78.9(76.2-82.5)	0.394	0.211
Energy	1819(1488-2203)	1936(1502-2319)	1489(1208-2092)	0.128	0.171
**Folate**	**416(326-581)**	**631(462-829)**	**720(526-1191)**	**<0.001**	**<0.001**
**Betaine**	**148(107-258)**	**199(152-335)**	**258(194-401)**	**0.001**	**<0.001**
**Choline**	**375(276-395)**	**475(438-490)**	**622(569-686)**	**<0.001**	**<0.001**

1. Discrete variables presented by number (%) and compared using the Chi-square test. Differences were considered to be statistically significant at P < 0.05.
2. Continuous variables were presented by median with the IQR (interquartile range), compared using the Kruskal–Wallis tests; trend test was performed using Jonckheere–Terpstra test. Differences were considered to be statistically significant at P < 0.05.
3. *Obesity was defined as at least one of the following features: BMI > 30kg/m2, Waist–Hip ratio >0.85 in female or >0.9 in male, visceral fat level >10 of inbody test, body fat percent >25% in male or >32 in female. Lean was define as none of above features.

Table S4: Clinical characteristics of female participants with PEMT rs 7946 GG genotype stratified by energy-adjusted dietary choline intake, with cutoff value of 448 mg/day

Variables	<448mg/day	>448mg/day	P value
No.	30	38	
Age*, yr	67(60-70)	63(55-67)	0.086
**BMI, kg/m^2**	**26.3(23.6-28.9)**	**23.8(21.8-26.9)**	**0.056**
Body Weght (kg)	61.9(53.7-66.4)	58.8(50.9-68.2)	0.521
**HS, No(%)**	**24(80)**	**18(47.4)**	**0.011**
Obesity, No(%)	26(86.7)	28(73.7)	0.236
Biochemical markers			
Cholesterol (mg/dL)	199(163-221)	201(177-231)	0.338
Triglycerides (md/dL)	118(66-163)	82.5(55.5-137)	0.170
LDL (mg/dL)	112(89.3-139)	116(89.0-141)	0.517
**HDL (mg/dL)**	**48.5(38.0-57.5)**	**52.0(43.0-65.0)**	**0.089**
AST (U/L)	24.0(22.0-38.0)	26.0(20.8-32.3)	0.701
ALT (U/L)	26.5(19.8-43.3)	27.0(19.0-42.3)	0.734

**CPK**	**120(80.3-158)**	**82.0(64.8-118)**	**0.009**
HbAlc (%)	6.00(6.00-6.20)	6.00(5.58-6.00)	0.098
Insulin (uIU/ml)	12.5(8.00-15.1)	10.5(7.49-13.1)	0.397
HOMA-IR	3.51(2.08-4.35)	2.68(1.88-3.70)	0.297
Dietary Nutrient Intake			
Energy, kcal	1292(1092-1807)	1440(1104-1913)	0.391
**Folate, µg**	**563(423-720)**	**765(507-996)**	**0.027**
**Betaine, mg**	**177(136-230)**	**258(174-357)**	**0.017**
**Total choline, mg**	**268(306-422)**	**548(498-605)**	**<0.001**
Plasma 1C Biomarker			
**RBC folate**	**365(289-450)**	**285(254-389)**	**0.084**
Plasm folate ng/ml	11.5(9.50-20.5)	10.4(6.75-16.3)	0.127
Plasma choline, µmol/L	11.4(9.50-13.1)	10.3(8.67-12.6)	0.271
Plasma betaine, µmol/L	44.8(34.8-49.9)	43.8(34.2-51.4)	0.800
Plasma Hcy, µmol/L	9.50(8.08-12.0)	8.99(7.05-11.0)	0.129

1. Continuous data was presented by median, 25th–75th percentile; discrete data was presented by no (%). Continuous variables were compared using the Mann–Whitney U test.; Discrete variables were compared using the Chi-square test. Differences were considered to be statistically significant at P < 0.05.
2. *Obesity includes BMI ≥30 kg/m2 or waist–hip ratio men ≥ 0.9, women ≥0.85, or visceral fat level ≥10 or body fat men ≥25 %, women ≥ 32 %.
